# Runs of Homozygosity and Quantitative Trait Locus/Association for Semen Parameters in Selected Chinese and South African Beef Cattle

**DOI:** 10.3390/ani12121546

**Published:** 2022-06-14

**Authors:** Mamokoma Cathrine Modiba, Khathutshelo Agree Nephawe, Jun Wang, Nompilo Hlongwane, Khanyisile Hadebe, Wenfa Lu, Bohani Mtileni

**Affiliations:** 1 College of Animal Sciences and Technology, Jilin Agricultural University, Changchun 130118, China; 205211926@tut4life.ac.za (M.C.M.); junwang2004@126.com (J.W.); 2 Joint Laboratory of Modern Agriculture Technology International Cooperation, Ministry of Education, Jilin Agricultural University, Changchun 130118, China; 3 Department of Animal Sciences, Tshwane University of Technology, Pretoria 0001, South Africa; nephaweka@tut.ac.za; 4 Agricultural Research Council, Biotechnology Platform, Pretoria 0110, South Africa; hlongwanen@arc.agric.za (N.H.); mdladlak@arc.agric.za (K.H.)

**Keywords:** ROH, inbreeding coefficient, quantitative trait locus/association and breeds

## Abstract

**Simple Summary:**

Genome-wide runs of homozygosity (ROH) are excellent in understanding population history, estimating genomic inbreeding, and deciphering genetic architecture of complex traits and diseases, as well as identifying genes of agro-economic traits. ROH are defined as continuous region on the chromosome where an individual is homozygous across the genome. This study identified distribution of ROH in the six selected beef cattle breeds, Chinese Simentaler (CSI), Belgian Blue (BEL), and South African Angus (ANG), Nguni (NGU), Bonsmara (BON), and Simentaler (SIM), using Bovine BeadChip markers. Furthermore, nine candidate genes, *CDF9*, *MARCH1*, *WDR19*, *SLOICI*, *ST7*, *DOP1B*, *CFAF9*, *INHBA*, and *ADAMTS1*, were suggested to be associated with semen QTL traits and reported moderate inbreeding in some breeds with high to low correlation inbreeding between breeds. The study findings will allow proper guidelines for breeder’s societies.

**Abstract:**

In this study, runs of homozygosity (ROH) and quantitative trait locus/association (QTL) for semen parameters in selected Chinese and South African beef cattle breed were estimated. The computed results showed 7516 ROH were observed between classes 0–5 Mb with no ROH observed in classes >40 Mb. Distribution of ROH showed high level of genomic coverage for ANG, NGU, CSI, and BEL breeds. Approximately 13 genomic regions with QTL were controlling sperm motility, sperm concentration, semen volume, sperm count, sperm head abnormalities, sperm tail abnormalities, sperm integrity, and percentage of abnormal sperm traits. Nine candidate genes, *CDF9*, *MARCH1*, *WDR19*, *SLOICI*, *ST7*, *DOP1B*, *CFAF9*, *INHBA*, and *ADAMTS1*, were suggested to be associated with above mentioned QTL traits. The results for inbreeding coefficient showed moderate correlation between FROH vs FHOM at 0.603 and high correlation between FROH 0–5 Mb 0.929, and lowest correlation for 0–>40 Mb 0.400. This study suggested recent inbreeding in CSI, BEL, ANG, BON, SIM, and NGU breeds. Furthermore, it highlighted varied inbreeding levels and identified QTL for semen traits and genes of association. These results can assist in implementation of genetic improvement strategies for bulls and provide awareness and proper guidelines in developing breeding programs.

## 1. Introduction

Breeding programs for cattle have been known to be widely implemented in a lot of countries in the world. Several countries have regarded breeding as an important aspect in beef cattle, while some countries have already implemented those programs. These programs include breeds like South African Bonsmara, an indigenous cross between European Shorthorn, Hereford, and Afrikaner [1], and the indigenous Southern African (South Africa, Eswatini, Namibia, Zimbabwe, Botswana, and Angola) Nguni breed a cross between *Bos taurus* and *Bos indicus*. The Simmentaler, a cross between German cattle and Swiss cattle is still one of the most utilized breeds in breeding programs. The Scottish Angus [2] has been in South Africa over a hundred years, even part of the South African Studbook. The Belgian blue originally from Belgium and is the first known Belgian breeds [3]. These programs lead to fast genetic progress, but they also lead to the accumulation of inbreeding via heavy impact of a few selected individuals [4]. Therefore, using genome-wide runs of homozygosity (ROH) is excellent in understanding population history and estimating genomic inbreeding, solving genetic architecture of complex traits and diseases, as well as identifying genes of agro-economic traits [5]. ROH can be defined as continuous regions on the chromosome where an individual is homozygous across the genome [5]. They occur when both haplotypes transmitted from parents are identical and inherited from a common ancestry [5]. An increase in homozygous loci and regions of homozygosity may be an indication of loss in genetic diversity [6], or population going through bottleneck.

A few studies have reported the negative impact of high homozygosity on fertility traits, including bull semen quality [7], calving rate [8], stillbirths, and dystocia [9]. A previous study [10] showed distinct differences in the length, quantity, and frequency of ROH between 11 Polish breeds as well as highlighting level of genomic inbreeding within breeds. The authors of [7] reported that longer ROH (>100 Mb) indicate recent inbreeding that has occurred within a population; however, shorter ROH may indicate ancient inbreeding that has occurred in population. Long ROH may persist in out bred individuals, due to unusual mutation, linkage disequilibrium (LD), and recombination rates at certain genomic locations [8]. The investigation of ROH in farm animals also suggested their importance contributions to complex traits [6]. Runs of homozygosity in American Holstein cattle revealed candidate genes associated with reproduction traits affecting fertility [11]. There are several factors which affects the quality of ROH calling rate; these include the marker density, their distribution across the genome, the quality of the genotype calling rates, and minor allele frequency [12]. Single nucleotide polymorphism provide information about both past and more recent demographic variations of a population [12], allowing a comparison of the degree of homozygosity among populations with varying degrees of isolation and inbreeding [5]. The authors [13] stated different approaches to estimating inbreeding at an individual and population level using genetic information. The objective of the study is to use distribution of ROH to identify inbred individuals in both South African and Chinese beef bulls and identify QTL for semen traits and their associated genes. This will assist in understanding population history and reveal genes linked to with semen traits. It will further show levels of inbreeding in commercial breeds, especially beef breeds [10].

## 2. Materials and Methods

### 2.1. Sampling, Genotyping and Quality Control

Approximately 144 semen samples were collected from South African and Chinese bulls, and genomic DNA was extracted from South African Bonsmara (n = 21), Angus (n = 22), Nguni (n = 28), and Simmental (n = 25), and from Chinese Belgian blue (n = 24) and Chinese Simmental (n = 24) breeds. Genotyping was conducted using Illumina Bovine 150 K BeadChip (Illumina, Inc, San Diego, CA, USA) for South African breeds and GGP Neogen Bovine 150 K (Neogen, Lansing, MI, USA) was used for Chinese breeds. All samples were processed with Genome Studio 2.0 software (Illumina, Inc, San Diego, CA, USA). Plink v1.07 [14] software was used for quality control to filter data according to the following criteria: (1) call frequency ≥ 90, (2) remove individuals with (MIND) ≤ 0.05, (3) SNPs with missingness (GENO) ≤ 0.05, (4) minor allele frequency (MAF) ≥ 0.05 and (5) Hardy–Weinberg equilibrium (HWE > 0.00001), and (6) samples that did not satisfy these criteria were excluded.

### 2.2. Estimation of Runs of Homozygosity (ROH)

Runs of homozygosity were estimated using PLINK v1.07 [14] following the criteria: (1) the minimum length was 1000 kb; (2) the proportion of homozygous overlap window was 0.05; (3) the minimum number of SNPs included in a ROH was 100; (4) the minimum SNP density was set to 50 kb/SNP; (5) the maximum gap between continuous homozygous SNPs was 1000 kb; and (6) a maximum of one SNPs with missing genotypes and up to one heterozygous genotype were allowed in a ROH. RStudio software was used, package “detectRUNS” version 0.9.6 was used to summarize ROH into five classes: (0–5 Mb), (5–10 Mb), (10–20 Mb), (20–40 Mb), and >40 for all six breeds. Runs of homozygosity were calculated per breed and ROH coverage in each chromosome was estimated as the sum of the total length of the chromosome covered by ROH of all individuals in a population. Furthermore, the percentage of SNPs present in ROH were calculated based on the frequency of a SNP in a ROH across individuals.

### 2.3. QTL and Genomic Regions in ROH

PLINK v1.07 [14] (Cambridge, MA, USA) was used to estimate the consensus regions across individuals, which represent ROH pools of overlapping and potentially matching segments. The software Bovine Quantitative Trait Locus (QTL) Analysis (QTL) [15] https://www.animalgenome.org.cgi-bin/qtldb/btsearch (accessed on 28 January 2022) [16] was used for information, gene information, and animal information. Then, National Center for Biotechnology Information (NCBI) https://www.ncbi.nlm.nih.gov (accessed on 27 March 2022) was used to confirm ID of the genes found from published QTL/association and functions. We then used KEGG [17] https://www.genome.jp/kegg-bin/showorganism?org=bta (accessed on 28 January March 2022) to identify the position and length of the genes.

### 2.4. Classification of Runs and Inbreeding Co-Efficient

Measuring homozygosity per individual was calculated following the method of [18].

$$\mathrm{FROH}=\sum \frac{\mathrm{LROH}}{\mathrm{LAUTO}}$$

LROH is the total length of ROH and LAUTO is the length of the autosomal genome [19]. The ROH length categories were as follows: >0 Mb, >5 Mb, >10 Mb, >20 Mb, and >40 Mb. Furthermore, inbreeding based on homozygous SNPs was determined using PLINK v1.07 software [14]. The inbreeding coefficient for an individual (FHOM) was computed. Correlations of the inbreeding coefficient for two methods were estimated using the Pearson correlation from Minitab ™ version 17.1.0 [20] (State College, PA, USA).

## 3. Results

### 3.1. Distribution of Runs of Homozygosity (ROH)

**Figure 1:**
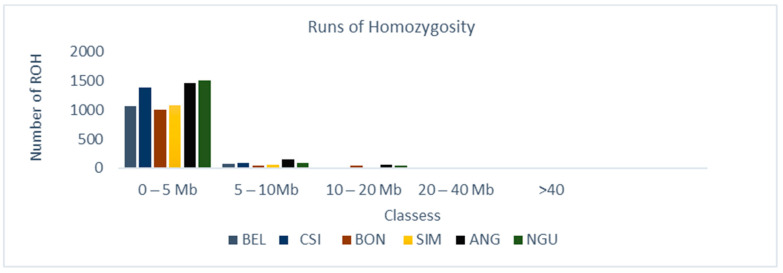
Classification of ROH were estimated per breed; each ROH length category and average per breed, represented from left to the right: Belgian Blue (BEL), Chinese Simmentaler (CSI), Bonsmara (BON), Nguni (NGU), South African Simmentaler (SIM), and Angus (ANG). All Breeds had the highest ROH between 0–5 Mb with the NGU and AGN showing the highest ROH 0–5 Mb on chromosomes 5, 6, 11, and 14 for SIM, and chromosomes 5, 7, and 14 for BON, respectively, in South African breeds.

**Figure 2:**
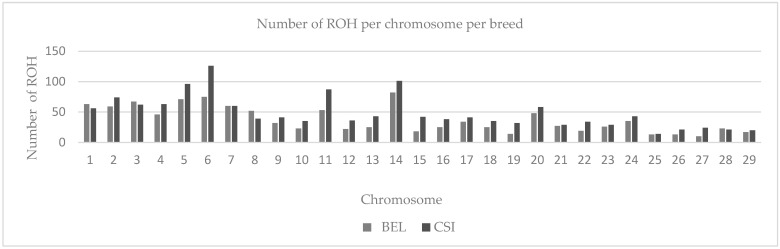
Number of ROH per chromosomes for Chinese CSI and BEL with significant ROH observed on chromosomes 5, 6, 11, and 14 for both breeds.

**Figure 3:**
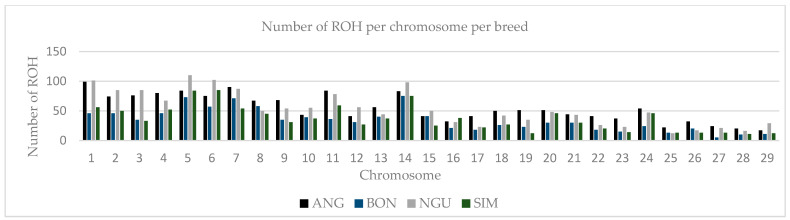
Number runs of homozygosity per chromosomes for South Africa BON, NGU, SIM, and ANG with significant ROH observed on chromosomes 1, 2, 3, 4, 5, 6, 7, 11, and 14, with NGU and ANG showing the most significant chromosome coverage.

In subsequent quality control steps, 117,042 SNPs and 144 individuals were retained in a genome 2.6 Gb. Approximately 7516 ROH were observed between classes 0–5 Mb, with the most ROH observed in NGU (1507), ANG (1456), CSI (1388), SIM (1083), BEL (1067), and BON (1006), shown on (Figure 1), respectively. In total, 514 ROH were observed between classes 5–10 Mb with ANG (149) and NGU (95) demonstrating the most ROH amongst all breeds. Fewer ROH were observed between classes 10–20 Mb, 20–40 Mb, and >40 Mb for all breeds. Distribution of ROH (Figure 2) coverage was observed on chromosomes 5, 6, 11, and 14 for CSI and BEL on chromosomes 1, 2, 3,5, 6, 7, and 14 for Chinese breeds. On behalf of South African breeds, ROH coverage (Figure 3) was observed on chromosomes 1, 2, 3, 4, 5, 6, 11, and 14 for NGU; chromosomes 1, 4, 7, 11, and 14 for ANG; chromosomes 5, 6, 11, and 14 for SIM; and chromosomes 5, 7, and 14 for BON.

### 3.2. The proportion of SNPs in ROH

**Figure 4:**
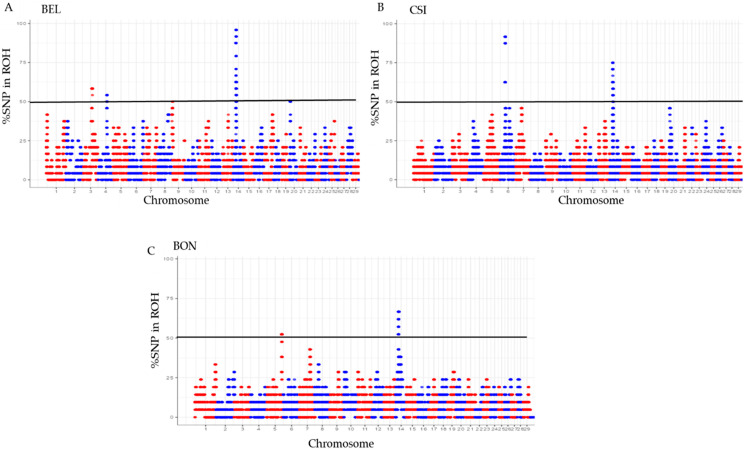

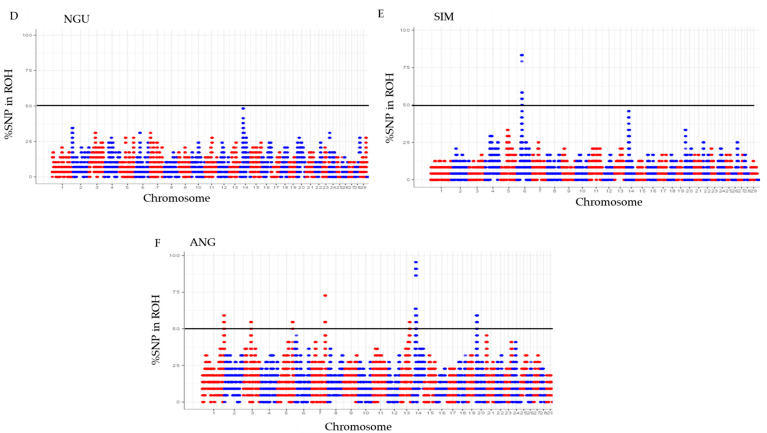
Manhattan plot for SNPs in ROH from the top to bottom (**A**) BEL, (**B**) CSI, and (**C**) BON, with BTA 14 having the most significant SNP in ROH for the three breeds. Manhattan plot for SNPs in ROH from the top to bottom (**D**) NGU, (**E**) ANG, and (**F**) SIM, with ANG having the most significant SNP in ROH on BTA 1,3, 5,7,13,14, and 20 compared to SIM and breeds.3.3. QTL and Identification of Candidate Genes within Runs of Homozygosity.

A Manhattan plot was used to compute the number of significant SNP in a ROH (Figure 4). The results revealed SNP in ROH across all individual breeds (Figure 4A) with BEL showing significant SNP in ROH on *BTA* 3, 4, and 14. (Figure 4B), CSI on *BTA* 6 and 14, (Figure 4C); for BON on *BTA* 6 and 14, (Figure 4F) ANG on *BTA* 1, 3, 5, 7, 13, 14, and 20, and (Figure 4E) for SIM, significant SNP were detected on *BTA* 6 and 14, (Figure 4D), with no significant SNP for NGU breeds. The results further highlighted most significant SNP were higher in all breeds on *BTA* 14, excluding NGU and SIM. The South African ANG had the most significant SNP in ROH based on *BTA* coverage. Further results supporting Manhattan plot are shown on (Table 1).

Runs of homozygosity length segments were used to detect QTL for semen traits using cattle QTL online database. QTL were identified for sperm motility, sperm concentration, semen volume, sperm count, sperm head abnormalities, sperm tail abnormalities, sperm integrity, and percentage of abnormal sperm, with the majority QTL suggested for sperm motility accounting for 80% of the results (Table 2). On *BTA* 6, the study revealed suggestive QTL for sperm motility, sperm head abnormalities, and sperm tail abnormalities located on the *WDR 19* gene. *MARCH1* gene also shared an association on *BTA* 6 to suggestive QTL for sperm count, semen volume, and sperm motility, with the majority of QTL on *BTA* 6 reported for Chinese CSI and South African SIM breeds covering 68 Mb of the genome position. Secondly on *BTA* 1 suggestive QTL for sperm concentration revealed an association to *CFAP9* gene and QTL for sperm motility and sperm count revealed an association to *DOP1B* gene, with suggestive QTL for sperm motility revealing an associated with *ADAMTS1* and *CRYZL1* gene. Other suggestive QTL for ANG breed were observed on *BTA 7* for sperm acrosome integrity rate, sperm motility and sperm count which revealed an association to *GDF9* gene. However, some of the suggestive QTL for percentage of normal sperm on BTA 13 and sperm motility on *BTA 3* showed no association to any gene for ANG breeds.

The South African BON on *BTA 5* revealed QTLs for sperm motility and it’s associated with *SLCO1C1* gene and BEL on *BTA 5* suggested QTL for sperm motility and revealed an association with *SLCO1C1* and *WDR 19* genes, also *INHBA* and *ST7* genes on *BTA 4* found an associated to sperm count. The results reported 13 genomic regions had identified QTL and their association; additionally, *BTA 14* had the greatest coverage of SNP in ROH in CSI, BEL, BON, and ANG, but there were no detection of QTL or association on those regions.

### 3.3. Inbreeding Coefficient FROH vs. FHOM and ROH Regions

**Figure 5:**
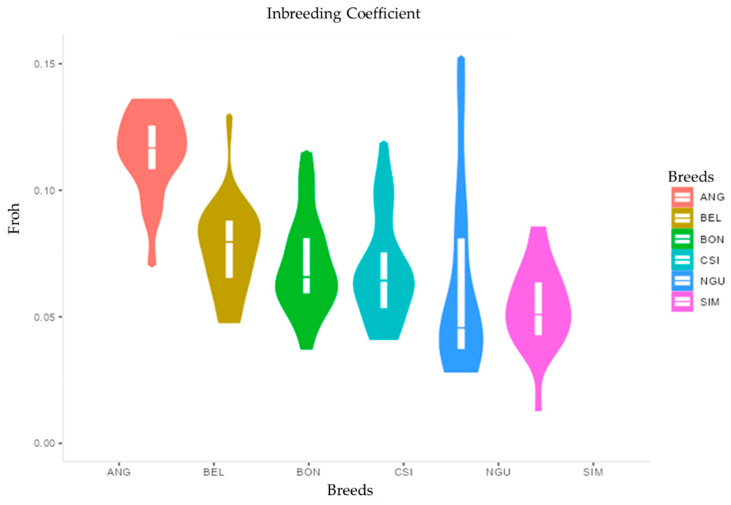
Inbreeding coefficient at a genomic level for all six breeds.

Lengths of ROH were further used to estimate inbreeding coefficient (FROH), shown on (Figure 5); inbreeding coefficient (FROH) was highest in NGU (0.16), followed by ANG (0.14), BEL (0.13), CSI (0.12), BON (0.11), and, finally, SIM (0.08). The Pearson correlation method was used to estimate the linear correlation coefficient between five classes and at a genomic level shown on (Table 3). Correlation estimated for FROH vs FHOM was 0.603, showing a significant moderate correlation. However, the highest correlation was observed between Froh _5 vs. Froh _0 at (0.92), and the lowest correlation was observed between Froh _40 vs. Froh _5 (0.40). This shows that the most correlation was observed the first classes (0–5 Mb).

## 4. Discussion

Inbreeding is known to negatively affects the reproductive performance of male animals [21], and when expressed at high levels can cause poor semen quality [22,23,24,25]. However, not all inbreeding reported is harmful; the authors of [26] reported that recent inbreeding is more harmful than ancient inbreeding due to selection decreasing the frequency of deleterious alleles over time. Many studies have explored the genome wide distribution of ROH and inbreeding depression in cattle populations [9,27,28] using high density Illumina BovineHD BeadChip microarrays. This includes studies such as [29], whose findings showed that ROH are frequent across all breeds (Angus, Belgian Blue, Charolais, Friesian, Hereford, Holstein, Holstein-Friesian, Limousin, and Simmental); however, difference in patterns of ROH and variation is based on breed origin and recent management. The authors of [10] reported the distinct differences in length, quantity, and frequency of ROH between breeds and levels of genomic inbreeding. The study highlighted higher levels of inbreeding observed in commercial breeds, especially beef breeds and identified a number of genes confirmed to influence production traits [10]. Homozygous regions of the genome [8] have been reported in different species, not only in cattle [19,30], but these regions have been used to quantify individual inbreeding in humans [18], goats [31], buffalo [32], sheep [33], and pigs [12]; based on results of ROH in these studies, it appears to be more accurate than traditional pedigree-based estimates [13]. In this study, more than 1000 Mb ROH were estimated in six selected Chinese and South African beef cattle breeds at a genomic length of 2.6 Gb, with the highest ROH observed in the NGU, ANG, BEL, CSI, SIM, and BON breeds. Furthermore, the highest ROH were observed between classes 0–5 Mb compared to the rest of the classes (5–10 Mb, 10–20 Mb and 20–40 Mb) with no ROH observed in >40 class. Similar results were reported by [29], who compared European cattle breeds to the European bison, with average ROH observed for Angus and Hereford, and the most ROH were observed for the European bison breeds between classes 1–5 Mb, suggesting limited recent inbreeding for Angus and Hereford, but reporting high levels of inbreeding for European bison. These results confirm earlier studies that reported population history involving a severe bottleneck [29]. Runs of homozygosity can be affected by demographic events [34], e.g., age, geography, breed history and origin, area of distribution, climate, introduction of breeds, environment, production system, etc.; the fact that breeds from this study are distributed in two geographically different environments might explain the inbreeding. The authors of [35] reported that inbred individuals are particularly sensitive to environment changes this can explaining most of the breeds in the study are inbred due to moving from one captive environment to another captive environment. Analysis at a genomic level revealed significant distribution of ROH within the selected six Chinese and South African beef cattle breeds. Chinese CSI and BEL had significant ROH observed on chromosomes 5, 6, 11, and 14 for both breeds. South Africa BON showed the highest ROH on chromosomes 5, 7, and 14, and SIM displayed the most ROH on chromosomes 5, 6, and 14. The highest genome coverage in ROH observed for ANG on chromosomes 1–6, 11, and 14, and NGU on chromosomes 1–3, 5–7, 11, and 14. The study further used length and number of ROH to identify SNPs in those ROH. The authors of [30] reported that greatest number of ROH per chromosomes was observed for chromosome 1 across all 867 animals, and ROH per chromosome tended to decrease with chromosome length. By identifying the number of SNPs in ROH, the study identified overlapping ROH that were above the threshold of 0.05. Chromosome 14 had the most SNPs above the threshold; still, it did not report any QTL or association to any candidate gene in all breeds. Additionally, no SNP in ROH were identified for NGU; possibly markers were standardized for commercial breeds, e.g., (Simentaler) however Nguni is an indigenous Southern African breed. In total, we reported 13 genomic regions that were associated to nine candidate genes (*CDF9*, *MARCH1*, *WDR19*, *SLOICI*, *ST7*, *DOP1B*, *CFAF9*, *INHBA*, and *ADAMTS1*). The authors of [36] reported that genomic regions located on *BTA 7, BTA 14, BTA 16,* and *BTA 18* were characterized by a high frequency of ROH occurrence and included important genes related to immune traits, muscularity, and ease of calving. The authors of [10] reported a similar region, *BTA 6*, was identified for Polish Red, Limousin, and Simmental breeds. *MARCH1* [19] revealed an association with QTL for sperm motility and semen volume on *BTA 6* in SIM. The *MARCH 1* gene was also reported by [37] to be significantly associated with semen production traits (semen volume per ejaculate, number of sperm per ejaculate, and number of motile sperm per ejaculate). Another gene associated with QTL for sperm motility revealed on *BTA 5* is *SLCO1C1* [17] for BON and BEL. *BTA 7* revealed the growth differentiation GDF9 gene was associated with QTL for sperm motility, sperm count, and sperm integrity. This was also reported by [38] who revealed significant association of *GDF9* with sperm quality traits in Holstein bulls. The results also stated that *GDF9* is involved in the initiation or maintenance of spermatogenesis; however, further verification is needed. WD repeat domain 19 (*WDR 19*) gene was detected on *BTA 5* and *BTA 6* for CSI showed an association to QTL sperm motility, sperm head abnormalities, and sperm tail abnormalities; this gene was reported to be associated to ejaculate volume, sperm concentration, sperm motility, and sperm head and tail anomalies [39]. On *BTA 4*, *ST7* gene was significant to QTLs for sperm count and sperm motility, this gene was also reported by [40] as the nearest genes for poor sperm motility. QTL associated with semen traits were identified and validated by previously published literature. This study highlighted genes within QTL regions of semen for beef bulls. Identification of the QTL regions associated with these traits provides the knowledge necessary to enrich these regions [35]. The authors of [41] reported on three QTL found to be related with abnormal sperm frequencies at a significant *p* < 0.01. The authors of [40] reported several candidate genes associated with sperm concentration, sperm motility and sperm volume in Holstein-Friesian populations.

The study further showed high to moderate correlation between classes Froh_0 −5 Mb and correlation at Froh_ > 40. Moderate correlation was observed between Froh vs Fhom selected breeds. Several authors reported on strong correction between inbreeding coefficient [9]. It should be underlined that the occurrence of ROH in an individual may be the result of inbreeding events, but they may also be present in outbreed populations as result of other phenomena.

## 5. Conclusions

The study identified distribution of ROH in the six selected Chinese and south African beef cattle breeds (CSI, BEL, ANG, NGU, BON, and SIM) using Bovine HD Bead Chip makers. The study also showed that ANG, NGU, and CSI showed the highest frequency and length of long ROH (0–5 Mb), indicating higher recent inbreeding in all breeds, including SIM. Several QTL and genes were related to semen traits (*CDF9*, *MARCH1*, *WDR19*, *SLOICI*, *ST7*, *DOP1B*, *CFAF9*, *INHBA*, and *ADAMTS1*). These genes can be used as target genes for future marker-assisted selection.

## Figures and Tables

**Table 1:** SNP position, length, chromosome, and number of SNPs ROH.

Breeds	Start SNP	End SNP	Start Position	End Position	BTA	nSNP
SIM	BovineHD0600010649	BovineHD0600010935	38474338	39921321	6	258
SIM	BovineHD0600010973	ARS-BFGL-NGS-99026	40064733	72930338	6	1491
CSI	BovineHD1400006792	BovineHD1400006937	23394002	23917569	14	60
CSI	BTB-01143619	BovineHD1400007578	26196375	26302589	14	21
CSI	BovineHD4100004545	BovineHD0600010837	38290032	39418286	6	183
BON	BovineHD1400006736	BovineHD1400006801	23240328	23407192	14	55
BON	BovineHD1400007096	BovineHD1400007272	24448641	25069487	14	107
BON	ARS-BFGL-NGS 100816	BovineHD0500032567	111909943	112748475	5	39
BEL	BovineHD1400001029	BovineHD0300021345	7162753	73321455	3	43
BEL	BTA-107777	BovineHD0300022023	73921609	75752848	3	77
BEL	BovineHD0500024941	BovineHD0500026993	87870382	95025828	5	295
BEL	BovineHD1400006790	BovineHD1400007272	233392546	25069487	14	265
BEL	BovineHD1400007377	BovineHD1400007531	25607730	26108646	14	117
BEL	BovineHD0400019201	BovineHD0400019430	6990155	70569432	4	17
ANG	BovineHD0100044669	Hapmap23088-BTA-151194	153400885	154349918	1	47
ANG	BovineHD1300018328	BovineHD1300018406	64228423	64621429	13	10
ANG	BovineHD1400006790	BovineHD1400006916	23392546	23831754	14	31
ANG	BovineHD1400007051	BovineHD1400007272	24315353	25069487	14	55
ANG	BovineHD1400007366	BovineHD1400007408	25480962	25583674	14	31
ANG	BovineHD1400007518	BovineHD1400007583	26051609	26938603	14	49
ANG	BovineHD1400007694	BovineHD1400024442	26700286	26938603	14	52
ANG	BovineHD2000001713	BovineHD2000001840	5497761	5839847	20	81
ANG	BovineHD0300015826	BovineHD0300015865	52418548	52539507	3	32
ANG	BovineHD0500030711	BovineHD0500030764	106905471	106988256	5	47
ANG	BovineHD0700027123	BovineHD0700027253	92797461	93307177	7	53

**Table 2:** Breeds, QTL, BTA, and gene of association.

Breed	QTL TRAIT	BTA	Gene
SIM	Sperm motility, Sperm head abnormalities (SPHAB) and Sperm tail abnormalities	6	WD repeat domain 19 (*WDR 19*)
	Semen volume, sperm motility and sperm count	6	*MARCHF1* membrane associated ring-CH-type finger 1 (*MARCH1*)
CSI	Sperm motility, Sperm head abnormalities (SPHAB) and Sperm tail abnormalities (SPTAB)	6	WD repeat domain 19 (*WDR 19*)
BON	Sperm motility (SPMOT)	5	WD repeat domain 19 (*WDR 19*)
	Sperm motility (SPMOT)	5	Solute carrier organic anion transporter family member 1C1 (*SLCOICI*)
BEL	Sperm motility (SPMOT)	5	Solute carrier organic anion transporter family member 1C1 (*SLCO1C1*) WD repeat domain 19 (*WDR 19*)
	Sperm count and sperm motility	4	suppression of tumorigenicity 7 (*ST7*) inhibin subunit beta A (*INHBA*)
ANG	Sperm motility and sperm count	1	DOP1 leucine zipper like protein B (*DOP1B*)
	Sperm concentration	1	1 cilia and flagella associated protein 91 *CFAP9*
	Sperm motility	1	crystallin zeta like 1 (*CRYZL1*) ADAM metallopeptidase with thrombospondin type 1 motif 1 (*ADAMTS1*)
	Percentage normal sperm	13	-
	Sperm motility	3	-
	Sperm acrosome integrity rate, sperm motility and sperm count.	7	growth differentiation factor 9 *GDF9*

**Table 3:** Correlations runs of homozygosity (ROH) between class category (0, 5, 10, 20, and >40 Mb), inbreeding coefficients in six different bulls.

Classes	Froh_Class_0	Froh_Class_5	Froh_Class_10	Froh_Class_20	Froh
Froh_Class_5	0.929				
Froh_Class_10	0.711	0.864			
Froh_Class_20	0.567	0.743	0.881		
Froh_Class_40	0.400	0.527	0.599	0.665	
Fhom					0.603
